# Latent profiles of active ageing and their association with oral health-related quality of life among rural older adults in China

**DOI:** 10.3389/fpubh.2026.1809594

**Published:** 2026-08-10

**Authors:** Xiaofang Song, Zuojuan Yin, Jianyi Bao, Shasha Li, Yashuang Sun, Canqi Ren, Mengxue Shi

**Affiliations:** 1 Department of Nursing, College of Medical Science, Huzhou Normal University, Huzhou, Zhejiang, China; 2 Department of Pulmonary and Critical Care Medicine, Shandong Provincial Hospital Affiliated to Shandong First Medical University, Jinan, Shandong, China

**Keywords:** active ageing, latent profile analysis, oral health-related quality of life, public health, rural older adults

## Abstract

**Background:**

Active ageing is a key strategy for addressing population ageing; however, the heterogeneous characteristics of active ageing among the rural older adults population have not yet been fully elucidated, and the association between active ageing and oral health-related quality of life (OHRQoL) has not been systematically explored.

**Methods:**

A cross-sectional survey was conducted among 598 rural older adults from April to October 2024. Latent profile analysis (LPA) was used to identify active ageing patterns; multinomial logistic regression was used to analyse predictors of category membership; and negative binomial regression was used to analyse the association between latent active ageing categories and OHRQoL.

**Results:**

Four distinct profiles of active ageing were identified: low-level (12.38%), medium-level with low social integration (17.56%), medium-level with low financial security (35.79%), and high-level (34.28%). Profile affiliation was significantly associated with nine factors, including age, educational attainment, and income (all *p* < 0.05). Negative binomial regression results showed that, with Profile 4 as the reference, Profile 1 (*β* = 0.581, 95% CI: 0.277–0.885, *p* < 0.001), Profile 2 (*β* = 0.561, 95% CI: 0.301–0.821, *p* < 0.001), and Category 3 (*β* = 0.480, 95% CI: 0.261–0.698, *p* < 0.001) all had significantly higher OHIP-14 total scores, suggesting that OHRQoL was more severely impaired in these three groups.

**Conclusion:**

The active ageing of rural adults in China exhibits heterogeneous characteristics and shows a significant gradient in oral health-related quality of life (OHRQoL). Differentiated intervention measures should be developed for different populations, and rural oral health services should be strengthened to improve their quality of life.

## 1 Introduction

The global population is rapidly ageing. By 2030, the World Health Organisation estimates that individuals aged 60 and above will comprise 16.7% of the global population (1). China has entered a profound ageing phase (2), particularly in rural areas, where older adults account for 23.81% (3). This proportion is expected to reach 37.7% by 2035 (4). This profound demographic shift exacerbates the strain on rural healthcare resources, the burden on social security systems, and the caregiving responsibilities within families. Rural older adults individuals face higher risks of comorbid chronic diseases (5), depression, and loneliness (6, 7), coupled with limited access to medical services (8). Against this backdrop, active ageing in rural areas has become a key strategy to address these challenges and improve the quality of life for older people, garnering increasing attention from policymakers and scholars (9).

Active ageing refers to the process by which older adults continuously participate in society and fully realise their personal potential, based on their own needs, aspirations, and abilities (10). Its core framework consists of three dimensions: “health, participation, and security” (11). This concept is influenced by multiple interacting factors, encompassing individual characteristics (such as age, gender, health status) (12, 13), lifestyle factors (such as physical exercise, sleep habits, dietary patterns) (14, 15), social environmental factors (16, 17), and economic and institutional safeguards (18). Existing evidence indicates that the level of active ageing among rural older adults in China is generally lower than in urban areas (19), particularly as reflected in lower social participation (20), insufficient older adults care security, and weak awareness of rights and interests (21). However, rural older populations exhibit significant internal disparities in health status, economic resources, and social support, and are not homogeneous; their patterns of active ageing are likely to present multiple subtypes. Therefore, identifying the heterogeneity of active ageing within this population is a necessary prerequisite for achieving precise allocation and efficient utilisation of older adults care resources.

The “health” dimension of active ageing encompasses not only physical function and psychological state but also oral health, a critical component (22). However, the Fourth National Oral Health Epidemiological Survey revealed that the periodontal health rate among older adults individuals aged 65–74 in China was only 9.3%, while the caries prevalence among permanent teeth reached 98% (23). Such high prevalence rates imply substantial dental restoration needs. Yet, access to denture rehabilitation services remains markedly inadequate in rural areas—a survey conducted in rural Yunnan showed a local restoration rate of only 33.66% (24). Dental loss or deficiency can lead to chewing difficulties and unclear speech, severely impairing oral function and reducing social interaction capabilities (25), ultimately resulting in a decline in oral health-related quality of life (OHRQoL). This indicator comprehensively reflects the overall impact of oral issues on an individual’s physiological, psychological, and social functions (26). Existing studies have confirmed that feeding difficulties caused by dental loss or deficiency are associated with reduced OHRQoL (27). Longitudinal studies further demonstrate that poor OHRQoL is significantly correlated with cognitive decline and frailty in older people (28, 29), which are precisely key barriers to active ageing. Nevertheless, current research has not systematically explored the relationships between OHRQoL and various dimensions of active ageing among rural Chinese seniors, nor has there been sufficient investigation from an individual-centred perspective into the heterogeneous patterns of active ageing and their association with OHRQoL, which is the core focus of this study.

Previous research on active ageing in older people has primarily employed variable-centred approaches (such as regression analysis) that focus on overall levels (18, 30). While these methods help identify general trends, their underlying assumption of homogeneity may mask significant heterogeneity within populations, making it difficult to reveal the diverse patterns of active ageing that may exist internally. From a life-course perspective, active ageing is a complex dynamic process shaped by multiple interacting factors, with each individual’s ageing trajectory being unique (31); this theoretical framework provides robust support for using individual-centred methods to capture heterogeneity. Rojo-Perez et al. (32) identified five subgroups of active ageing among the older adults population through cluster analysis; in China, Guo et al. (33) used latent profile analysis to classify community-dwelling seniors into three types: low-active ageing, moderate-active ageing-high-mindfulness wisdom, and high-active ageing-comprehensive. Individual-centred latent profile analysis (LPA) enables natural classification based on individuals’ responses across multiple indicators, revealing distinct internal subgroups with unique characteristics (32). However, no study has extended this method to rural older adults in China, given their significant environmental, cultural, and socioeconomic differences, which likely result in active ageing patterns markedly distinct from those observed in urban populations (31).

Therefore, this study employs the potential profile analysis method to identify potential subtypes of active ageing among rural older adults populations in China and to explore the associations between these subtypes and oral health-related quality of life (OHRQoL). By combining an individual-centred analytical approach with a dental health perspective, this study aims to deepen our understanding of the heterogeneity of active ageing and provide a basis for targeted health promotion and support service strategies in rural areas. The objectives of this study are as follows: (i) To identify potential subtypes of active ageing among rural older adults and describe their demographic characteristics; (ii) To investigate predictive factors influencing subtype assignment in active ageing; (iii) To examine the associations between different potential categories of active ageing and oral health-related quality of life (OHRQoL).

## 2 Methods

### 2.1 Study design

This study used a cross-sectional design and adhered to STROBE guidelines for reporting. The study was conducted in rural areas of Huzhou City, Zhejiang Province, from April to October 2024.

### 2.2 Participants

Inclusion criteria: (i) aged 60 years or older; (ii) permanent rural residence for ≥6 months. Exclusion criteria: (i) individuals who are deaf, blind, have aphasia, or cannot communicate effectively due to other physical conditions; (ii) individuals diagnosed with mental illness or cognitive impairment by a clinician.

### 2.3 Sampling methods and bias control measures

This study employed convenience sampling, with Huzhou City in Zhejiang Province serving as the sampling area, and was conducted from April to October 2024. To minimise sampling bias, the following measures were implemented: First, sampling sites were selected across multiple townships within the five administrative districts of Huzhou City, covering villages from diverse geographical locations and economic development levels, avoiding concentration at single or easily accessible locations; second, within each village, investigators, with assistance from primary healthcare workers, invited eligible older adults individuals who met inclusion criteria rather than selecting only those who were easily accessible or highly cooperative.

During the data collection phase, to minimise measurement and response bias, all investigators (master’s degree candidates in nursing) underwent standardised training before the survey and adhered to established procedures. The questionnaire was administered via face-to-face structured interviews: participants with normal vision and adequate literacy skills completed it independently; for those with visual impairments or limited literacy, investigators read the questions word-for-word without any paraphrasing or suggestive guidance. Before the survey began, investigators thoroughly explained the study objectives, procedure, voluntary participation, confidentiality, and potential benefits. Participants signed a written informed consent form after full comprehension and retained the right to withdraw at any time without justification. Each questionnaire took approximately 20 min to complete, and upon completion, each participant received a small gift valued at approximately $5 as a token of appreciation.

### 2.4 Sample size

According to the sample size calculation formula for cross-sectional studies: $n={\left(\frac{u_{\alpha }/2\sigma }{\delta }\right)}^{2}$ (34) this study used survey data on active ageing and its influencing factors from the previous literature (35). The parameters were set as follows: *α* = 0.05, *σ* = 18.09, and *δ* = 1.6. Substituting these values into the formula yielded a required sample size of approximately 491 participants. Meanwhile, considering the methodological requirements of the primary analytical approach in this study—Potential Profile Analysis (LPA)—regarding sample size, and following the recommendations of Sinha et al. (36) (which indicate that model fit statistics achieve higher accuracy and stability when the sample size exceeds 500), as well as accounting for potential invalid responses during questionnaire administration, a total of 610 questionnaires were distributed. After data cleaning, 12 invalid responses were excluded (exclusion rate: 2.0%), leaving 598 valid responses for statistical analysis and an effective response rate of 98.03%. The final valid sample size was 598 cases, meeting all aforementioned statistical criteria.

### 2.5 Measures

#### 2.5.1 General information questionnaire

A questionnaire including age, education, marital status, living conditions, monthly income, self-rated health status, number of chronic diseases, re-employment intention, farming situation, daily housework duration, intergenerational care, frequency of child visits, medical insurance, endowment insurance, tooth loss, and denture use.

#### 2.5.2 Chinese version of the active ageing scale for rural older adults (active ageing scale, AAS)

Active Ageing Scale (AAS), modified by Li et al., was used to assess the level of active ageing among rural older adults (35). The scale consists of 36 items across seven dimensions and is scored on a 4-point Likert scale (36–144), with higher scores indicating greater active ageing. The cut-off scores were 36–72 (low to moderate), 73–109 (moderate), and 110–144 (moderate to high) (33). The original scale had a Cronbach’s alpha coefficient of 0.824. In this study, the scale’s overall Cronbach’s alpha was 0.948.

#### 2.5.3 Oral health impact profile (OHIP-14) scale

Oral Health Impact Profile-14 (OHIP-14) scale to assess OHRQoL. The scale was simplified by Slade based on the OHIP-49 (37) and later sinicised by Xin et al. (38). The Chinese version consists of 14 items across four dimensions, scored on a 5-point Likert scale (0–56), with higher scores indicating poorer OHRQoL. The original scale had a Cronbach’s alpha of 0.930 (39). In this study, the overall Cronbach’s alpha was 0.891.

### 2.6 Statistical analysis

Data entry was performed using Epidata 3.1. Quantitative variables were presented as means ± standard deviations, and categorical variables as frequencies and percentages. Latent profile analysis (LPA) was conducted in Mplus 8.3 using the seven dimensions of the Active Ageing Scale (AAS) as indicator variables. The number of profiles was progressively increased to estimate model parameters. Model fit was evaluated using the following criteria: (1)Akaike Information Criterion (AIC), Bayesian Information Criterion (BIC), and Sample-size Adjusted Bayesian Information Criterion(SABIC): lower values indicated better model fit (40); (2) Entropy index: values ≥ 0.80, especially those approaching 1.0, indicated high classification accuracy (>90%) (41); (3) Lo-Mendel-Rubin likelihood ratio test (LMRT) and Bootstrap Likelihood Ratio Test (BLRT): significant *p*-values (*p* < 0.05) suggested that the model with k profiles provided a significantly better fit than the model with k−1 profiles (42). When model fit indices provided inconsistent recommendations, the final model was selected based on a comprehensive evaluation of statistical criteria, theoretical interpretability, sample size, and practical relevance.

Data analysis was performed using SPSS 26.0, with potential LPA-identified categories serving as grouping variables. One-way ANOVA, chi-square tests, or Fisher’s exact test were used to compare differences in general demographic characteristics across categories. Statistically significant variables from the one-way analysis were included in multinomial logistic regression models to identify potential predictors of category assignment. To investigate the association between active ageing potential categories and OHRQoL, the OHIP-14 total score was used as the dependent variable (continuous variable). After normality and over-discreteness tests, the data exhibited a skewed distribution and over-discreteness; thus, a negative binomial regression model was adopted. Independent variables were the potential categories (with Profile 4 as the reference). Model 1 did not adjust for covariates; Model 2 adjusted for age, education level, number of chronic diseases, reemployment intention, monthly income, self-rated health status, marital status, intergenerational care, and daily household labour duration. Results are presented as regression coefficients (*β*) and 95% confidence intervals (CI).

## 3 Results

### 3.1 General characteristics of participants

Participants’ ages ranged from 60 to 95 years, with a mean age of 71.64 ± 7.40 years. Among them, 60.37% were female, 68.06% had at least a primary school education, 73.58% were married, and 21.07% lived alone. Regarding income, 41.97% earned between 1,000 and 2000 per month. 47.99% reported better self-rated health, and 36.12% had two or more chronic conditions. Additionally, 51.34% had no intention of re-employment, and 53.01% were not engaged in farming. Concerning daily activities, 13.38% spent ≤0.5 h on housework, 55.52% participated in intergenerational care, 73.41% experienced tooth loss, and 38.13% used dentures. Most participants had medical and endowment insurance. Other characteristics are detailed in Table 1.

**Table 1:** General characteristics of participants (*n* = 598).

Variable	*N*	%/M ± SD
Age		
60–69	244	40.80
70–79	261	43.64
≥80	93	15.55
Sex		
Male	237	39.63
Female	361	60.37
Educational level		
Secondary school and above	169	28.26
Primary school	238	39.80
illiteracy	191	31.94
Marital status		
Married	440	73.58
Widowed/divorced/unmarried	158	26.42
Living condition		
Live with spouse	261	43.65
Live with children	211	35.28
Live alone	126	21.07
Monthly income (yuan)		
>2000	172	28.76
1,000–2000	251	41.97
<1,000	175	29.26
Self-rated health status		
Better	287	47.99
General	177	29.60
Poor	134	22.41
Number of chronic diseases		
0	155	25.92
1	227	37.96
≥2	216	36.12
Re-employment intention		
Yes	291	48.66
No	307	51.34
Farming situation		
Yes	281	46.99
No	317	53.01
Daily housework time(hour/day)		
0.5 h or less	80	13.38
More than 0.5 h, up to 2 h	161	26.92
More than 2 h, up to 4 h	215	35.95
More than 4 h	142	23.75
Intergenerational care		
Yes	332	55.52
No	266	44.48
Frequency of child visits		
At least once a day	142	23.75
At least once a week	149	24.92
Half a month or at least once a month	200	33.44
Less than once a month	107	17.89
Medical insurance		
Yes	577	96.49
No	21	3.51
Endowment insurance		
Yes	483	80.77
No	115	19.23
Tooth loss		
Yes	439	73.41
No	159	26.59
Denture use		
Yes	228	38.13
No	370	61.87
Active ageing scale(score, *M ± SD*)	—	94.59 ± 19.27
Oral Health Impact Profile(score, *M ± SD*)	—	22.04 ± 10.28

### 3.2 Descriptive analysis of active ageing and OHRQoL in rural older adults

The active ageing score of rural older adults was (94.59 ± 19.27). Among the seven dimensions, the highest mean score was for self-care ability (3.08 ± 0.72), and the lowest was for active contribution to society (2. 15 ± 0.82). The OHIP-14 score was (22.04 ± 10.28). Among the five dimensions, the highest mean score was for pain and discomfort in the mouth itself (5.16 ± 0.13), and the lowest was for decreased independence (1.31 ± 0.84). Details are shown in Table 2.

**Table 2:** Scores of active ageing and oral health impact among rural older adults (*n* = 598).

Dimension	Number of items	Maximum value	Minimum value	Score (M ± SD)	Item mean score
Self-care ability	7	28	7	21.56 ± 5.06	3.08 ± 0.72
Active learning and integration into society	8	32	8	18.86 ± 5.76	2.36 ± 0.72
Active contribution to society	4	16	4	8.60 ± 3.26	2.15 ± 0.82
Development of spiritual wisdom	5	20	5	14.23 ± 3.35	2.85 ± 0.67
Establishment of financial security	4	16	4	8.79 ± 3.56	2.20 ± 0.89
Maintaining a healthy lifestyle	5	20	5	13.41 ± 3.37	2.68 ± 0.67
Inheriting filial piety and leading by example	3	12	3	9.14 ± 2.31	3.05 ± 0.77
Active ageing scale	36	144	36	94.59 ± 19.27	—
Decreased independence	5	19	0	6.54 ± 4.21	1.31 ± 0.84
Psychological discomfort	3	12	0	5.16 ± 3.26	1.72 ± 1.09
Discomfort in body functions	3	12	0	5.05 ± 3.10	1.68 ± 1.03
Pain and discomfort in the mouth itself	3	12	0	5.29 ± 2.88	5.16 ± 0.13
Oral health impact profile-14	14	50	0	22.04 ± 10.28	—

### 3.3 Latent profile analysis of active ageing among rural older adults

**Figure 1:**
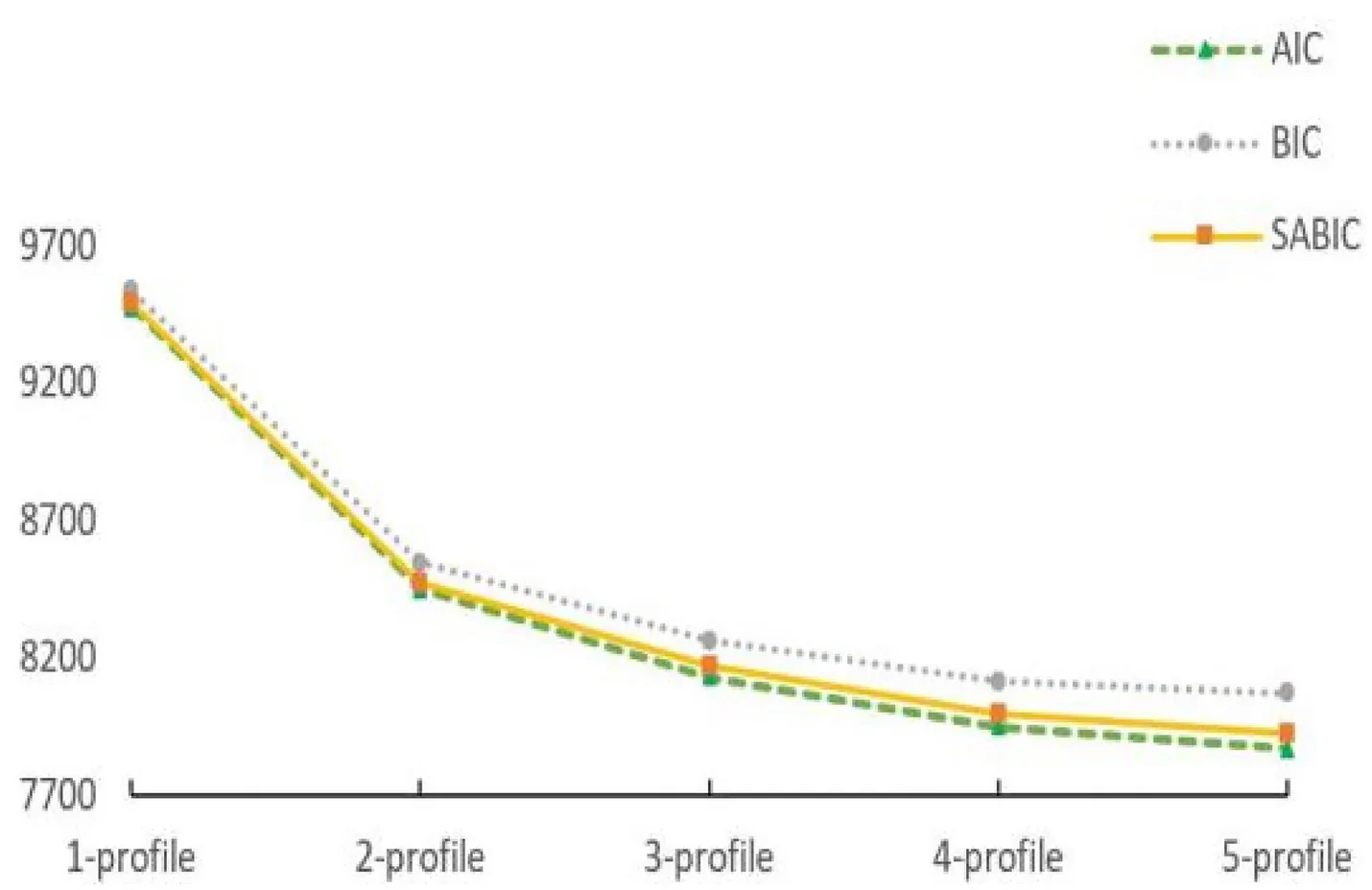
Latent profile model fit index steep slope map.

Using scores across the seven dimensions of active ageing as indicators of observation, five potential profile models (profiles 1–5) were sequentially fitted. Model fit metrics are detailed in Table 3, while trends in AIC, BIC, and aBIC are shown in Figure 1. As the number of categories increased, all information criterion values continued to decline; however, the rate of decrease slowed significantly when the number rose from four to five profiles, indicating limited improvement from additional profiles. The entropy values for all models (profiles 2–5) exceeded 0.80, with profile 4 achieving the highest (0.836), demonstrating excellent classification accuracy. Likelihood ratio tests revealed that both LMRT and BLRT for the profile 4 model were statistically significant (*p* < 0.001).

**Table 3:** Fitting statistics for a latent profile model of active ageing in rural older adults (*n* = 598).

Model	Loglikelihood	AIC	BIC	SABIC	Entropy	*p*-value		Profile probability
						LMR-LRT	BLRT	
1-profile	−4723.641	9475.282	9536.792	9492.346	N/A	N/A	N/A	1
2-profile	−4202.776	8449.552	8546.211	8476.367	0.825	<0.001	<0.001	0.38/0.62
3-profile	−4035.711	8131.422	8263.229	8167.988	0.799	0.0158	<0.001	0.25/0.41/0.34
4-profile	−3936.216	7948.432	8115.389	7994.750	0.836	<0.001	<0.001	0.12/0.18/0.36/0.34
5-profile	−3889.037	7870.073	8072.179	7926.142	0.827	0.186	<0.001	0. 17/0. 12/0.31/0.21/0.19

N/A, not applicable; AIC, Akaike information criterion; BIC, Bayesian information criterion; SABIC, sample-size adjusted Bayesian information criterion; LMR-LRT, Lo-Mendell–Rubin’s adjusted likelihood ratio test; BLRT, bootstrap likelihood ratio test. The 4-profile model (bold) was selected as the optimal model.

In contrast, LMRT for the profile 5 model was not significant (*p* = 0.186), suggesting that the profile 5 model did not show a substantial improvement over the profile 4 model. Sample proportions for each category in the profile 4 model were 12.38%, 17.56%, 35.79%, and 34.28%, all exceeding the 5% threshold; average posterior classification probabilities for the four categories ranged from 0.876 to 0.949, indicating reliable classification results and clear category distinctions. Specifically, although the profile 3 model also exhibited an acceptable fit, its LMRT was statistically significant (*p* < 0.001), confirming a marked improvement from profile 3 to profile 4. Moreover, the additional category in the profile 5 model accounted for less than 5% of the total sample and lacked practical interpretative significance. Thus, the profile 4 model achieves an optimal balance between statistical fit and theoretical simplicity, and this study ultimately identifies it as the optimal classification framework for active ageing among rural older adults populations.

**Figure 2:**
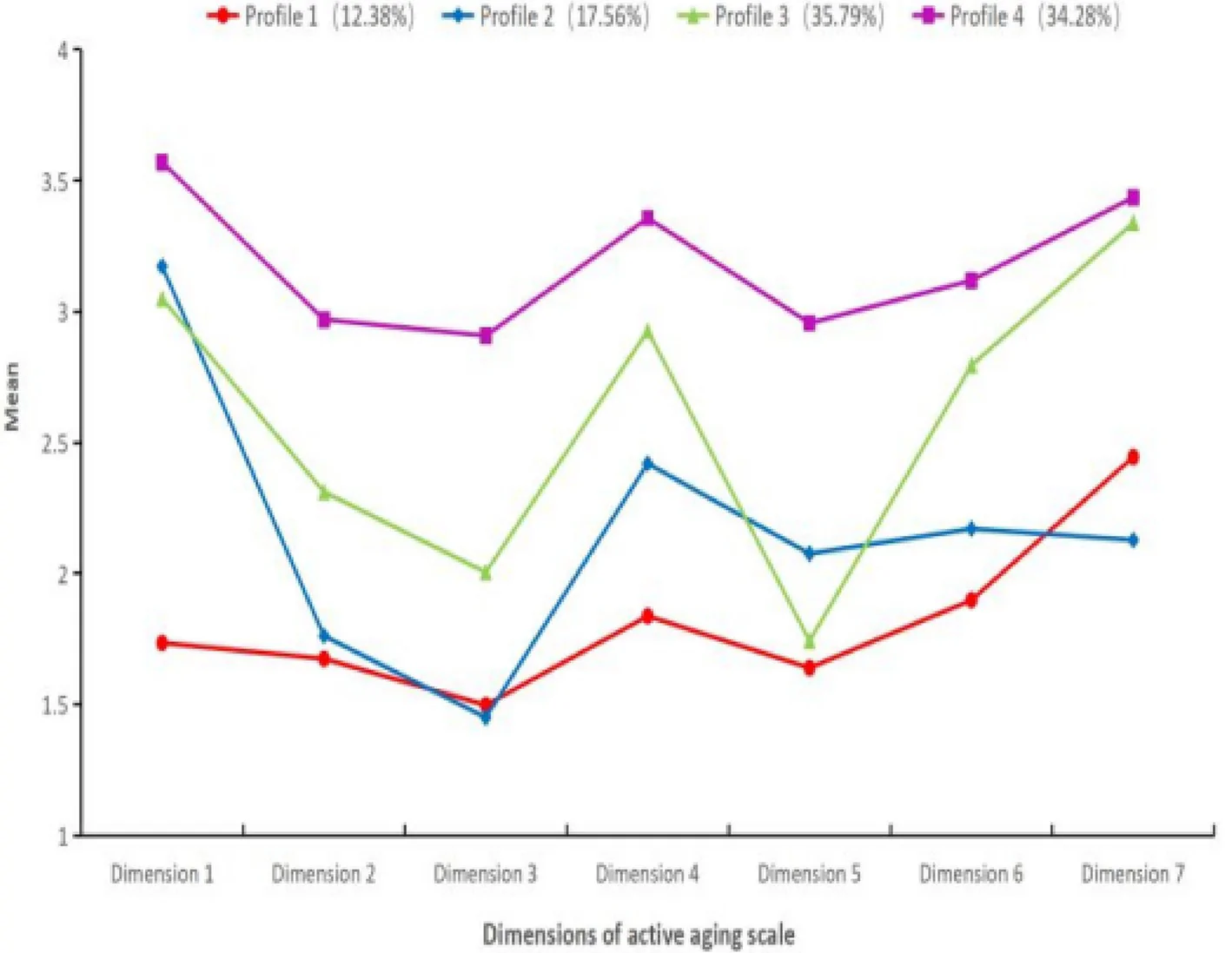
Four latent profiles of active ageing among rural older people. Active ageing scale consists of 7 dimensions. Dimension 1: Self-care ability; Dimension 2: Active learning and integration into society; Dimension 3: Active contribution to society; Dimension 4: Development of spiritual wisdom; Dimension 5: Establishment of financial security; Dimension 6: Maintaining a healthy lifestyle; Dimension 7: Inheriting filial piety and leading by example.

The performance of the four potential categories in the overall active ageing score, along with their scores across each dimension, is shown in Table 4 and Figure 2. Profile 1 (63.80 ± 10.50) as “low-level active ageing”; Profile 2 (79.28 ± 8.76) and Profile 3 (93.04 ± 6.78) as “medium-level active ageing”; and Profile 4 (115.18 ± 8.18) as “high-level active ageing.” shows the average scores of these four profiles across the seven dimensions, plotted in a line graph (Figure 2). Profile 1, with a generally low score, accounted for 12.38% (*n* = 74) and was named “low-level active ageing.” Profile 2, with low scores in active learning and integration into society and active contribution to society, accounted for 17.56% (*n* = 105) and was named “medium-level active ageing with low social integration.” Profile 3, with the lowest score in establishment of financial security, accounted for 35.79% (*n* = 214) and was named “medium-level active ageing with low financial security.” Profile 4, with high scores across all dimensions, accounted for 34.28% (*n* = 205) and was named “high-level active ageing”.

**Table 4:** Descriptive analyses of active ageing scale scores for four latent profiles (M ± SD, *n* = 598).

Model	*N*	%	Self-care ability	Active learning and integration into society	Active contribution to society	Development of spiritual wisdom	Establishment of financial security	Maintaining a healthy lifestyle	Inheriting filial piety and leading by example	Active ageing scale
Profile1	74	12.38	11.91 ± 2.89	13.43 ± 4.32	6.01 ± 1.84	9.14 ± 2.20	6.51 ± 2.25	9.45 ± 2.94	7.35 ± 2.12	63.80 ± 10.50
Profile2	105	17.56	22.25 ± 3.31	13.78 ± 3.99	5.70 ± 1.63	11.99 ± 2.29	8.44 ± 3.19	10.78 ± 2.74	6.34 ± 1.46	79.28 ± 8.76
Profile3	214	35.79	21.23 ± 3.60	18.44 ± 3.86	8.00 ± 2.39	14.62 ± 2.35	6.81 ± 2.22	13.94 ± 2.30	10.00 ± 1.69	93.04 ± 6.78
Profile4	205	34.28	25.04 ± 2.58	23.86 ± 4.18	11.64 ± 2.48	16.81 ± 1.95	11.86 ± 3.08	15.64 ± 2.49	10.32 ± 1.61	115.18 ± 8.18

### 3.4 Multinomial logistic regression of active ageing profiles among rural older adults

As shown in Table 5, significant differences (*p* < 0.05) in active ageing profiles among rural older adults were observed across age, education, monthly income, marital status, living condition, reemployment intention, self-rated health status, number of chronic diseases, intergenerational care, and daily housework duration. The above statistically significant variables were included in multiple logistic regression models, and the results are presented in Table 6.

**Table 5:** Bivariate analysis of different latent profiles of active ageing among rural older adults [*n*(%)].

Variable	Profile 1 (*n* = 74)	Profile 2 (*n* = 105)	Profile 3 (*n* = 214)	Profile 4 (*n* = 205)	*F*/*X*^2^	*p*-value
Age	74.46 ± 7.45	74.46 ± 7.45	71.75 ± 7.47	69.37 ± 6.88	14.009^c^	<0.001
Sex					0.647^a^	0.886
Male	32(43.2)	43(41.0)	83(38.8)	79(38.5)		
Female	42(56.8)	62(59.0)	131(61.2)	126(61.5)		
Educational level					53.543 ^a^	<0.001
Secondary school and above	11(14.9)	25(23.8)	39(18.2)	94(45.9)		
Primary school	30(40.5)	43(41.0)	95(44.4)	70(34. 1)		
illiteracy	33(44.6)	37(35.2)	80(37.4)	41(20.0)		
Marital status					13.701^a^	0.003
Married	45(60.8)	69(65.7)	169(79.0)	157(76.6)		
Widowed/divorced/ unmarried	29(39.2)	36(34.3)	45(21.0)	48(23.4)		
Living condition					15.688^a^	0.016
Live with spouse	37(50.0)	46(43.8)	86(40.2)	92(44.9)		
Live with children	16(21.6)	30(28.6)	91(42.5)	74(36. 1)		
Live alone	21(28.4)	29(27.6)	37(17.3)	39(19.0)		
Monthly income (yuan)					95.983^a^	<0.001
>2000	11(14.9)	22(21.0)	32(15.0)	107(52.2)		
1,000–2000	34(45.9)	40(38. 1)	105(49. 1)	72(35. 1)		
<1,000	29(39.2)	43(41.0)	77(36.0)	26(12.7)		
Self-rated health status					64.257^a^	<0.001
Better	17(23.0)	47(44.8)	100(46.7)	123(60.0)		
General	17(23.0)	43(41.0)	68(31.8)	49(23.9)		
Poor	40(54. 1)	15(14.3)	46(21.5)	33(16. 1)		
Number of chronic diseases					25.678 ^a^	<0.001
0	12(16.2)	14(13.3)	63(29.4)	66(32.2)		
1	27(36.5)	38(36.2)	84(39.3)	78(38.0)		
≥2	35(47.3)	53(50.5)	67(31.3)	61(29.8)		
Re-employment intention					73.844^a^	<0.001
Yes	20(27.0)	66(62.9)	68(31.8)	137(66.8)		
No	54(73.0)	39(37. 1)	146(68.2)	68(33.2)		
Farming situation					2.853^a^	0.415
Yes	35(47.3)	57(54.3)	96(44.9)	93(45.4)		
No	39(52.7)	48(45.7)	118(55. 1)	112(54.6)		
Daily housework duration(hour/day)					25.517^a^	0.002
0.5 h or less	18(24.3)	15(14.3)	26(12. 1)	21(10.2)		
More than 0.5 h, up to 2 h	17(23.0)	34(32.4)	52(24.3)	58(28.3)		
More than 2 h, up to 4 hours	16(21.6)	27(25.7)	85(39.7)	87(42.4)		
More than 4 h	23(31. 1)	29(27.6)	51(23.8)	39(19.0)		
Intergenerational care					31.257^a^	<0.001
Yes	27(36.5)	42(40.0)	140(65.4)	123(60.0)		
No	47(63.5)	63(60.0)	74(34.6)	82(40.0)		
Frequency of child visits					6.657^a^	0.673
At least once a day	24(32.4)	26(24.8)	50(23.4)	42(20.5)		
At least once a week	13(17.6)	29(27.6)	55(25.7)	52(25.4)		
Half a month or at least once a month	22(29.7)	32(30.5)	73(34. 1)	73(35.6)		
Less than once a month	15(20.3)	18(17. 1)	36(16.8)	38(18.5)		
Medical insurance					5.893^b^	0.099
Yes	68(91.9)	102(97. 1)	210(98. 1)	197(96. 1)		
No	6(8. 1)	3(2.9)	4(1.9)	8(3.9)		
Endowment insurance					7.487^a^	0.058
Yes	57(77.0)	76(72.4)	179(83.6)	171(83.4)		
No	17(23.0)	29(27.6)	35(16.4)	34(16.6)		
Tooth loss					3.850^a^	0.278
Yes	59(79.70)	82 (78. 10)	153 (71.50)	145 (70.70)		
No	15(20.30)	23 (21.90)	61(28.50)	60(29.30)		
Denture use					6.133^a^	0.105
Yes	27 (36.50)	51(48.60)	79 (36.90)	71 (34.60)		
No	47 (63.50)	54 (51.40)	135 (63. 10)	134 (65.40)		
OHIP-14 (score, M ± SD)	28.91 ± 7.67	27.73 ± 8.80	24.07 ± 7.78	14.51 ± 9.50	80.078^c^	<0.001

a Chi-square test.

b Fisher exact test.

c ANOV.

**Table 6:** Multinomial logistic regression analysis of active ageing profiles among rural older adults.

Variable	Profile 2 vs. profile 1 (ref)				Profile 3 vs. profile 1 (ref)				Profile 4 vs. profile 1 (ref)			
	*β*	*p*-value	OR	95% CI	*β*	*p*-value	OR	95% CI	*β*	*p*-value	OR	95% CI
Age	−0.010	0.689	0.990	0.942–1.040	−0.037	0.116	0.964	0.921–1.009	−0.051	0.041	0.951	0.906–0.998
Educational level (ref: illiteracy)												
Secondary school and above	0.315	0.518	1.370	0.528–3.554	0.167	0.712	1.182	0.487–2.866	1.527	<0.001	4.602	1.865–11.358
Primary school	0.425	0.259	1.529	0.731–3.200	0.308	0.361	1.361	0.703–2.632	0.668	0.076	1.951	0.933–4.080
Number of chronic diseases (ref: ≥ 2)												
0	−0.360	0.454	0.698	0.272–1.792	0.966	0.019	2.627	1.170–5.896	1.112	0.012	3.039	1.283–7.199
1	−0.023	0.950	0.977	0.476–2.006	0.578	0.092	1.783	0.911–3.491	0.674	0.068	1.963	0.952–4.044
Re-employment intention (ref: No)												
Yes	1.159	0.001	3.186	1.566–6.482	−0.334	0.331	0.716	0.365–1.405	1.069	0.002	2.913	1.463–5.798
Monthly income (yuan) (ref: <1,000)												
>2000	0.063	0.897	1.065	0.411–2.762	−0.135	0.775	0.874	0.346–2.207	2.005	<0.001	7.428	2.895–19.058
1,000–2000	−0.315	0.413	0.730	0.343–1.552	0.046	0.893	1.047	0.533–2.056	0.795	0.047	2.214	1.011–4.848
Self-rated health status (ref: poor)												
Better	1.778	<0.001	5.917	2.498–14.018	1.626	<0.001	5.082	2.400–10.763	1.799	<0.001	6.045	2.706–13.504
General	1.785	<0.001	5.958	2.502–14.185	1.402	<0.001	4.064	1.909–8.652	1.149	0.008	3.155	1.355–7.342
Marital status (ref: widowed/divorced/unmarried)												
Married	0.504	0.288	1.655	0.653–4.192	1.367	0.002	3.922	1.685–9.125	0.718	0.136	2.051	0.798–5.270
Living condition (ref: live alone)												
Live with spouse	−0.016	0.976	0.984	0.343–2.822	−0.290	0.556	0.748	0.285–1.966	0.001	0.998	1.001	0.341–2.942
Live with children	0.322	0.530	1.380	0.505–3.765	0.762	0.106	2.142	0.851–5.391	0.464	0.372	1.591	0.574–4.408
Intergenerational care (ref: no)												
Yes	0.072	0.838	1.075	0.538–2.147	1.113	0.001	3.044	1.625–5.700	0.872	0.011	2.391	1.219–4.688
Daily housework duration (hour/day) (ref: 0.5 h or less)												
More than 4 h	0.177	0.721	1.193	0.452–3.151	0.296	0.502	1.344	0.567–3.189	0.178	0.719	1.195	0.452–3.155
More than 2 h, up to 4 h	0.603	0.253	1.828	0.650–5.140	1.215	0.010	3.370	1.343–8.453	1.452	0.005	4.274	1.566–11.662
More than 0.5 h, up to 2 h	0.677	0.181	1.969	0.729–5.316	0.687	0.135	1.988	0.807–4.898	0.849	0.095	2.336	0.864–6.319

The “Medium-level active ageing with low social integration group”: Rural older adults individuals with intentions for reemployment (OR = 3.18695% CI: 1.566–6.482, *p* = 0.001), those self-reporting moderate health status (OR = 5.95895% CI: 2.502–14.185, *p* < 0.001), and those self-reporting good health status (OR = 5.91795% CI: 2.498–14.018, *p* < 0.001) were more likely to belong to this group. The “medium-level active ageing with low financial security group”: Rural older adults individuals who did not suffer from chronic diseases (OR = 2.62795% CI: 1.172–5.888, *p* = 0.019), self-rated general health status (OR = 4.06495% CI: 1.854–8.908, *p* < 0.001), self-rated good health status (OR = 5.08295% CI: 2.358–10.957, *p* < 0.001), were married (OR = 3.92295% CI: 1.663–9.248, *p* = 0.002), participated in intergenerational care (OR = 3.04495% CI: 1.555–5.960, *p* = 0.001), or spent 2–4 h daily on household chores (OR = 3.370,95% CI: 1.340–8.476, *p* = 0.010) were more likely to fall into the moderate-level active ageing – low financial security group. The “High-level active ageing group”: Rural older adults individuals with a secondary education or higher (OR = 4.60295% CI: 2.096–10.105, *p* < 0.001), no chronic diseases (OR = 3.03995% CI: 1.274–7.247, *p* = 0.012), intention to re-enter the workforce (OR = 2.91395% CI: 1.489–5.698, *p* = 0.002), monthly income ≥1,000 yuan (1,000–2,000 yuan: OR = 2.21495% CI: 1.011–4.847, *p* = 0.047;>2,000 yuan: OR = 7.42895% CI: 3.210–17.189, *p* < 0.001), self-rated moderate health status (OR = 3.15595% CI: 1.350–7.371, *p* = 0.008), self-rated good health status (OR = 6.04595% CI: 2.630–13.896, *p* < 0.001), and daily household chores duration of 2–4 h (OR = 4.27495% CI: 1.548–11.803, *p* = 0.005) were more likely to belong to the high-level active ageing group.

### 3.5 Relationship between active ageing profiles and OHRQoL in rural older adults

Using the OHIP-14 total score as the dependent variable, negative binomial regression was conducted to examine the association between potential active ageing categories and oral health-related quality of life (OHRQoL), with results presented in Table 7. Using Profile 4—the category with the highest level of active ageing—as the reference group, Model 1 (without adjustment for any covariates) revealed that the OHIP-14 total scores of Profile 1 (*β* = 0.68995% CI: 0.417–0.961, *p* < 0.001), Profile 2 (*β* = 0.64895% CI: 0.407–0.888, *p* < 0.001), and Profile 3 (*β* = 0.50695% CI: 0.309–0.703, *p* < 0.001) were significantly higher than those of Profile 4, indicating that the OHRQoL was markedly poorer in these three categories with lower levels of active ageing.

**Table 7:** Negative binomial regression analysis on the association between latent profiles of active ageing and OHRQoL.

Variable	Model 1 (unadjusted)			Model 2 (Adjusted)		
	*β*(SE)	95%CI	*p*-value	β(SE)	95%CI	*p*-value
Potential profile (reference: profile 4)						
Profile 1	0.689(0.1385)	0.417 ~ 0.961	<0.001	0.581(0.1553)	0.277 ~ 0.885	<0.001
Profile 2	0.648(0.1228)	0.407 ~ 0.888	<0.001	0.561(0.1325)	0.301 ~ 0.821	<0.001
Profile 3	0.506(0.1004)	0.309 ~ 0.703	<0.001	0.480(0.1116)	0.261 ~ 0.698	<0.001
Age	—	—	—	0.006(0.0065)	−0.007 ~ 0.019	0.348
Educational level	—	—	—	0.061(0.0574)	−0.051 ~ 0.174	0.285
Number of chronic diseases	—	—	—	0.010(0.0554)	−0.098 ~ 0.119	0.850
Re-employment intention	—	—	—	−0.015(0.0915)	−0.194 ~ 0.164	0.871
Monthly income	—	—	—	0.003(0.0395)	−0.075 ~ 0.080	0.944
Self-rated health status	—	—	—	0.027(0.0565)	−0.084 ~ 0.138	0.630
Marital status	—	—	—	0.054(0.1010)	−0.144 ~ 0.252	0.594
Intergenerational care	—	—	—	0.128(0.0890)	−0.047 ~ 0.302	0.151
Daily housework duration	—	—	—	0.002(0.0424)	−0.081 ~ 0.085	0.956

The dependent variable is the total score of the OHIP-14 scale, where higher scores indicate poorer oral health-related quality of life (OHRQoL); β denotes the regression coefficient, SE represents the standard error, and CI stands for the confidence interval; Profile 4 constitutes the group with the highest level of active ageing and serves as the reference group; Model 1 does not adjust for covariates, whereas Model 2 adjusts for age, education level, number of chronic diseases, willingness to return to employment, monthly income, self-rated health status, marital status, intergenerational care, and daily household labor duration.

After adjusting for potential confounding factors such as age, education level, number of chronic diseases, reemployment intention, monthly income, self-reported health status, marital status, intergenerational care, and daily household labor duration (Model 2), the aforementioned associations remained statistically significant: The total OHIP-14 scores in Profile 1 (*β* = 0.58195% CI: 0.277–0.885, *p* < 0.001), Profile 2 (*β* = 0.56195% CI: 0.301–0.821, *p* < 0.001), and Profile 3 (*β* = 0.48095% CI: 0.261–0.698, *p* < 0.001) were significantly higher than those in Profile 4. Among these, Profile 1 (the low-active ageing group) exhibited the highest *β* value, indicating the most severe impairment in OHRQoL. In Model 2, none of the included covariates was statistically significant (all *p* > 0.05).

## 4 Discussion

This study is the first to integrate potential profile analysis of active ageing with a dental health perspective, systematically identifying four potential categories of active ageing among rural older adults in China and revealing gradient differences in oral health-related quality of life (OHRQoL) across these categories. The effect size for the better OHRQoL group in the high-level active ageing category was significantly larger than that of traditional predictors such as education and income, and this association remained significant after adjustment for confounding factors. This finding empirically elevates oral health from a “local health issue” to a “strongly associated factor of active ageing,” expanding the theoretical scope of the “health dimension” of active ageing and providing new evidence-based insights for targeted health promotion strategies for rural older adults populations.

The four potential profile categories identified in this study—“Low-Level Active ageing Group,” “Moderate-Level Active ageing with Low Social Integration Group,” “Moderate-Level Active ageing with Low Financial Security Group,” and “High-Level Active ageing Group”—differ from the active ageing profiles identified by Guo et al. (33) among urban community seniors in both the number of classifications and category characteristics. This discrepancy may stem from two factors: first, the resource and development disparities resulting from the urban–rural dichotomy create a more complex social environment for rural older adults individuals, exhibiting distinct features compared to their urban counterparts (43); second, differences in measurement tools, as this study employed assessment instruments better suited to the unique characteristics of the rural older adults population. In summary, these variations in classification outcomes across different research contexts not only reflect the heterogeneity of older adults populations but also underscore the importance of conducting context-specific, group-differentiated studies.

Based on the aforementioned classification, this study further elucidates the characteristic differences among various subtypes of active ageing among rural older adults. The “low-level active ageing group” exhibits typical features, including advanced age, low education levels, and a high burden of chronic diseases, and achieves the lowest scores across all seven dimensions. This pattern may be linked to functional decline associated with ageing (44) and insufficient capacity for active ageing behaviours due to limited educational attainment (45), underscoring the urgency of implementing targeted interventions for this population. The “high-level active ageing group” scored higher than all other groups and above the sample mean across all dimensions, representing the ideal state of active ageing in rural settings. These findings align with those reported by Wu et al. (46) in older adults individuals with disabilities, who similarly identified a “well-adapted group” characterised by high, comprehensive scores across all dimensions of active ageing, confirming the stability of this high-functioning profile across different older adults populations.

It is noteworthy that the group with moderate levels of active ageing accounts for 53.35% of the total sample, yet shows significant internal differentiation: approximately two-thirds belong to the “low financial security category,” while one-third fall into the “low social integration category.” Although these groups share similar overall scores, their disadvantaged dimensions differ markedly—the older adults in the “moderate active ageing–low social integration” category score lower on proactive learning ability, social participation, and social contribution. This group often suffers from chronic comorbidities and engages predominantly in agricultural labour, which limits their opportunities for social engagement (47, 48). Despite strong reemployment intentions, they struggle to translate these into actual participation due to livelihood pressures, creating a “willing but unable” dilemma (49). In contrast, those in the “moderate active ageing–financial security” category exhibit low income levels and limited reemployment aspirations, with their economic security scores closely resembling those of the “low-level active ageing” group, reflecting the core challenges faced by rural seniors: although possessing moderate potential for active ageing, their weak economic foundations and inadequate social security systems severely constrain their capacity for active ageing (50). Therefore, identifying these disparities and incorporating them into policy design and resource allocation is crucial for promoting balanced and positive ageing (51).

To deepen understanding of the mechanisms underlying these category classifications, this study further analyzed the predictive factors for each subtype. Regarding structural resource factors, older adults individuals with a junior high school education or higher and a monthly income of ≥2,000 yuan were more likely to be in the high-level group. At the same time, those with older ages were more prone to belong to the low-level group, supporting the resource accumulation theory (52): higher education enhances health literacy and social adaptability (45); income security ensures material safety and participation opportunities (53), whereas age advantage correlates with better health outcomes and social engagement (54), collectively shaping the level of active ageing. In terms of family and health factors, intergenerational caregiving exhibits a dual “benefit-overload” effect: independently caring seniors benefit from family support networks (55), whereas married caregivers face dual pressures of caregiving burdens and financial strain, making them more likely to fall into the “moderate-low financial security” category (56). Regarding health indicators, older adults individuals reporting good self-rated health without chronic diseases are more likely to enter the high-level group, reflecting the synergy between subjective health perception and objective health status; however, some individuals with “fair” self-rated health still face economic difficulties due to inadequate pension coverage and limited employment opportunities (14). Regarding individual behavioural characteristics, 48.7% of older adults individuals expressed a desire to reemploy and were more likely to be in the moderate-to-low or high social integration groups. This willingness represents a key manifestation of active ageing (57). However, some individuals with such intentions ended up in the low social integration group due to limited opportunities for participation, highlighting the need to strengthen rural employment support systems. Daily household chores performed for 2–4 h can promote active ageing by improving physical and mental health, reducing the risk of frailty, and slowing cognitive decline (58, 59). Nevertheless, excessive household tasks may lead to fatigue and encroach on social time; thus, targeted guidance on balancing household duties and leisure activities should be provided to those in the “moderate-to-low financial security” group.

Interestingly, this study revealed a significant gradient distribution of the OHIP-14 total score across four categories of active ageing: the lower the level of active ageing, the more severe the impairment in oral health-related quality of life (OHRQoL). This association remained statistically significant after adjusting for confounding factors such as age, education level, number of chronic diseases, reemployment intention, monthly income, self-rated health status, marital status, intergenerational care, and daily household labour duration (all *p* < 0.001), indicating that active ageing serves as an independent determinant of oral health-related OHRQoL among rural older adults. This gradient relationship can be explained through three mechanisms. Physiologically-functional-wise, oral health directly impacts nutritional intake efficiency (60); individuals with tooth loss or chewing dysfunction tend to consume soft foods, leading to insufficient intake of proteins, dietary fibre, and trace elements, which subsequently compromises muscle strength, immune function, and cognitive reserves—key physiological foundations of active ageing. Psychosocially, oral issues (pain, edentulism, halitosis) significantly affect older adults individuals’ appearance confidence and social engagement (61); those with poor OHRQoL may avoid social interactions due to concerns about appearance or breath odour, resulting in reduced social participation. Rouxel’s research further confirms a strong correlation between low OHRQoL and loneliness in older people (62), with loneliness serving as a core barrier to social engagement and aligning precisely with the characteristics of the “moderately active ageing–low social integration” group. From an economic and resource perspective, the costs of oral disease treatment and denture restoration impose significant financial burdens on rural older adults individuals, limiting access to oral healthcare services (63). Furthermore, diminished Oral Health-Related Quality of Life (OHRQoL) further restricts their ability to participate in labour and contribute to society—this explains the dual disadvantages of the “low financial security group” in terms of both economic stability and OHRQoL.

These three dimensions interconnect, forming a multidimensional framework for how oral health influences active ageing, demonstrating that OHRQoL serves not only as a reflection of oral health but also as a bridge connecting physiological health with psychosocial well-being (28, 60, 62). By incorporating OHRQoL as an integrated metric into our analytical framework, this study expands the conceptual scope of the “health dimension” within active ageing: oral health affects not only nutritional intake but also, through its impact on social interactions and psychological states, indirectly influences multiple aspects of active ageing. In Model 2, traditional oral health predictors such as age, education level, and monthly income did not reach statistical significance (*p* > 0.05). Yet, the latent category of active ageing remained significantly associated with OHRQoL. This finding suggests that the comprehensive state of active ageing may be a more robust predictor of oral health-related quality of life among older adults compared to individual demographic or socioeconomic factors. In essence, the core value of this study lies not in identifying “which single factor affects oral health,” but in revealing that the overall state of active ageing experienced by older adults better explains variations in OHRQoL than any isolated factor. This conclusion supports implementing oral health interventions for older people from a multidimensional, holistic perspective rather than a fragmented one.

These findings provide clear targets for oral health interventions: different categories of actively ageing seniors exhibit distinct oral health issues that require tailored approaches. For the “low-level active ageing group,” oral health examinations should be incorporated into rural basic public health services, with annual dental screenings conducted by village physicians, free basic oral care provided to low-income and severely disadvantaged seniors, and mobile medical units regularly deployed to villages for those with mobility challenges. For the “moderate-to-low social integration group,” integrated oral health and social interaction education should be offered at village clinics, using peer education to reduce social stigma, while linking oral health check-ups with community senior activities to bridge the gap between oral health and social integration. For the “moderate-to-low financial security group,” efforts should focus on including certain dental restoration procedures within the New Rural Cooperative Medical Scheme’s reimbursement scope and on promoting self-care protocols for village-level oral health kits to maximise oral health benefits at minimal cost. The core strategy for the “high-level active ageing group” is to train them as “senior oral health advocates,” establish regular follow-up mechanisms, and leverage their exemplary role. Given that rural seniors often avoid urban dental facilities due to transportation barriers, financial concerns, or healthcare-seeking habits, it is imperative to strengthen the rural oral health service system. This includes enhancing basic dental care capabilities at village clinics, complemented by mobile medical services; strengthening oral health education; improving accessibility and trust in rural dental care; and ultimately promoting overall levels of active ageing among rural seniors.

The present study has the following limitations. First, self-reported questionnaires may introduce recall bias, affecting data accuracy. Future research should consider qualitative or mixed-methods approaches to achieve a more comprehensive understanding. Second, the cross-sectional design limits causal inference and trend analysis. Longitudinal studies are needed to explore active ageing dynamics over time. Third, the study’s focus on Huzhou City, Zhejiang Province, may limit the generalizability of the findings. Expanding the sample to multiple regions would enhance representativeness and facilitate multicenter investigations.

## 5 Conclusion

This study categorises active ageing among rural older adults into four potential groups: “Low-level active ageing group,” “Moderate-level active ageing with low social integration group,” “Moderate-level active ageing with low financial security group,” and “High-level active ageing group.” Given the multidimensional nature of active ageing and significant heterogeneity within these groups, targeted interventions tailored to each category’s specific needs are essential to provide more effective support services for rural seniors and enhance their quality of life in later years. Furthermore, the study reveals that active ageing categories are independent determinants of oral health-related quality of life (OHRQoL) among rural older adults; lower levels of active ageing are associated with greater OHRQoL impairment, providing empirical evidence to inform the development of oral health intervention strategies from an active ageing perspective.

## Data Availability

The raw data supporting the conclusions of this article will be made available by the authors, without undue reservation.
